# Entrepreneurs’ Courage, Psychological Capital, and Life Satisfaction

**DOI:** 10.3389/fpsyg.2019.00789

**Published:** 2019-04-05

**Authors:** Kristi Bockorny, Carolyn M. Youssef-Morgan

**Affiliations:** ^1^School of Business, Northern State University, Aberdeen, SD, United States; ^2^College of Business, Bellevue University, Bellevue, NE, United States

**Keywords:** entrepreneurship, courage, psychological capital, hope, efficacy, resilience, optimism, life satisfaction

## Abstract

Entrepreneurship involves numerous risks and uncertainties. Positive psychological resources such as courage, as well as confidence, hope, optimism, and resilience (collectively referred to as psychological capital), can be valuable for entrepreneurs. This study examines that relationship between entrepreneurs’ courage, psychological capital, and life satisfaction. Results show that entrepreneurs’ courage is related to their life satisfaction, even after accounting for various characteristics of the entrepreneur (demographics and human capital) and the venture (venture size and survival). Moreover, psychological capital fully mediates the relationship between courage and life satisfaction. This is the first study to investigate courage empirically in the context of entrepreneurship, and one of a few studies to apply PsyCap in the entrepreneurial context. It is also the first study to jointly examine courage, PsyCap, and life satisfaction. Implications for future research and practice are discussed.

## Introduction

This study explores the relationships between courage, psychological capital (PsyCap), and life satisfaction in the context of entrepreneurship. PsyCap is conceptualized and tested as a mediator of the relationship between entrepreneurs’ courage and their life satisfaction. This is the first study to empirically investigate courage, and one of a few studies to apply PsyCap, in the entrepreneurial context.

Peterson and Seligman (2004) define courage as a character strength that promotes the exercise of will to achieve goals, even when faced with opposition. A courageous decision contains an element of risk, the freedom to act, and a purposeful outcome (Shelp, 1984; Woodard, 2004; Rate, 2007; Rate et al., 2007).

Entrepreneurs often deal with plenty of risks and uncertainties. Courage can be a critical psychological resource for entrepreneurs. Naughton and Cornwall (2006) consider courage to be the most important quality for successful entrepreneurs. Without courage, an individual may never follow through with an entrepreneurial idea or leave the perks and certainties of typical employment to pursue entrepreneurship.

PsyCap is a higher-order construct defined as “an individual’s positive psychological state of development and is characterized by: (1) having confidence (self-efficacy) to take on and put in the necessary effort to succeed at challenging tasks; (2) making a positive attribution (optimism) about succeeding now and in the future; (3) persevering toward goals and, when necessary, redirecting paths to goals (hope) in order to succeed; and (4) when beset by problems and adversity, sustaining and bouncing back and even beyond (resiliency) to attain success” (Luthans et al., 2015, p. 2). Hope, efficacy, resilience, and optimism work together to produce results greater than each of the individual resources. The underlying mechanism or common thread linking PsyCap’s resources is that they all share a “positive appraisal of circumstances and probability for success based on motivated effort and perseverance” (Luthans et al., 2007, p. 550).

Entrepreneurs’ PsyCap is positively related to venture performance (Hmieleski and Carr, 2008), authentic leadership (Jensen and Luthans, 2006) and wellbeing (Hmieleski and Carr, 2007; Roche et al., 2014). Interestingly, entrepreneurs with higher PsyCap have been found to experience lower, rather than higher levels of stress and anxiety than the average population. This highlights the importance of PsyCap in attracting and sustaining entrepreneurship efforts (Baron et al., 2016).

Entrepreneurial success can take on many forms. Financial return is certainly an indicator of success. However, an average entrepreneur typically has a lower income than fulltime employed counterparts (Hamilton, 2000). Many individuals tend to pursue entrepreneurship, not only as a job, but as a lifestyle. Entrepreneurship fulfills many social and psychological needs. Thus, an entrepreneur’s satisfaction is an important global indicator of success.

Life satisfaction is defined as “a global assessment of a person’s quality of life according to his chosen criteria” (Shin and Johnson, 1978, p. 478). Life satisfaction occurs when an individual cognitively assesses his or her current life against a preconceived subjective standard and believes to have met or exceeded that standard (Pavot and Diener, 1993). An individual may choose money, prestige, family, or work, as important life domains for determining life satisfaction (Diener et al., 1985). Kim-Prieto et al. (2005) posit that subjective wellbeing is determined by positive appraisals and memories. Since venture success is subjective to each entrepreneur (Naughton and Cornwall, 2006), life satisfaction is an important and meaningful entrepreneurship outcome.

Courage can enhance entrepreneurs’ PsyCap in several ways. One of the most effective ways to build PsyCap efficacy is through mastery experiences (Bandura, 1997). Courageous entrepreneurs are likely to be more confident than their less courageous counterparts who never dare to try, take risks, and embrace uncertainty to build those valuable experiences. Courage can also promote PsyCap hope and optimism. A courageous entrepreneur may perceive a risky but feasible path around a setback as a possibility with a “can do” and “will do” attitude. Finally, entrepreneurs will most likely face adversities and setbacks. Resilient individuals function well under environmental stress and uncertainty (Rutter, 1987; Klohnen, 1996). Thus, courage can promote the PsyCap resources of hope, efficacy, resilience, and optimism, and the following is hypothesized.

Hypothesis 1: Entrepreneurs’ Courage Is Positively Related to Their PsyCap

Research supports a positive relationship between PsyCap and satisfaction with various life domains including work, health, relationships, and life in general (Luthans et al., 2007; Youssef and Luthans, 2007; Avey et al., 2010, 2011; Luthans et al., 2013). According to Kim-Prieto et al. (2005), factors that affect one’s subjective evaluations of life (i.e., life satisfaction) include actual life events, affective reactions to events, selective memory and recall, and global evaluations of one’s life in general. Because PsyCap involves positive appraisals of one’s circumstances and chances of success, individuals with higher levels of PsyCap are more likely to experience life satisfaction within life domains. Thus, the following is hypothesized.

Hypothesis 2: Entrepreneurs’ PsyCap Is Positively Related to Their Life Satisfaction

In line with Rate’s (2007) description of courage as containing an element of risk, the freedom to act, and a purposeful outcome, an entrepreneur who has courage faces the risk(s) involved, and exercises the freedom to act and move forward with the venture, with an eye toward the desired outcome of a successful and fulfilling venture. The entrepreneur’s subjective assessment of risk, the freedom to act and the outcome will all affect the entrepreneurship venture, and in turn, the entrepreneur’s overall life satisfaction. Thus, the following is hypothesized.

Hypothesis 3: Entrepreneurs’ Courage Is Positively Related to Their Life Satisfaction

Courage can help an individual face the risk and take the leap to make the dream a reality. A courageous entrepreneur will determine the level of risk associated with the goals for the business and possible outcomes. In turn, this courage to start and pursue an entrepreneurial venture can help the entrepreneur build confidence, find a different path if the initial one is no longer viable (hope), persevere and bounce back from setbacks (resilience), and maintain a positive outlook (optimism). In turn, PsyCap’s positive appraisals and agentic, intentional approach can promote satisfaction with the entrepreneurial experience and lifestyle in general. In contrast, without PsyCap, the entrepreneur may just give up and attribute failure to individual shortcomings, which can lead to dissatisfaction. Thus courage can contribute to entrepreneurs’ life satisfaction, both directly and indirectly through leveraging PsyCap, and the following is hypothesized.

Hypothesis 4: Entrepreneurs’ PsyCap Mediates the Relationship Between Their Courage and Life Satisfaction

## Methods

A survey was sent to all entrepreneurs who filed a new registration with the Secretary of State in a Midwestern state 2 years prior to the study (*N* = 3540). Carland et al. (1984) defined a successful entrepreneurial venture as one that has survived for 2 years. Thus, the 2-year mark was used as an indicator of venture survival. 152 entrepreneurs completed the survey.

Courage was measured using the 12-item Courage Measure (Norton and Weiss, 2009). PsyCap was measured using the 12-item Psychological Capital Questionnaire (Norman et al., 2010). The Satisfaction with Life Scale was used to measure entrepreneur’s life satisfaction (Diener et al., 1985).

We also included age, race, gender, degree, education, work experience, and entrepreneurial experience as control variables due to their systematic relationships with the study variables (Kim-Prieto et al., 2005; Avey, 2014), particularly in the entrepreneurship context (Prabhu, 1999; Platman, 2003; Boyd, 2005; Fairlie, 2007; Hmieleski et al., 2015). Importantly, venture survival [Carland et al.’s (1984) 2-year survival criterion] was controlled in order to account for the objective success or failure of the venture. Finally, number of employees was controlled as a proxy for venture size.

## Results

Descriptive statistics, correlations, and Cronbach’s alphas are represented in Table 1. Correlations between courage, PsyCap, and life satisfaction were all significant, supporting hypotheses 1–3.

**Table 1:** Descriptive statistics, zero-order correlations, and Cronbach’s alphas.

Study Variables	Mean	SD	1	2	3	4	5	6	7	8	9	10	11	12
1. Age	44.8553	11.40025	**N/A**											
2. Race [Caucasian (94%) = 1, Other = 0]	N/A	N/A	-0.106	**N/A**										
3. Gender [Female (43%) = 1, Male = 0]	N/A	N/A	-0.094	-0.0121	**N/A**									
4. Degree [Business (40%) = 1, Other = 0]	N/A	N/A	-0.128	0.035	0.052	**N/A**								
5. Years of education	17.3816	2.72573	0.097	-0.232**	0.055	0.048	**N/A**							
6. Work experience	24.7763	12.46356	0.845**	-0.047	-0.167*	-0.088	0.015	**N/A**						
7. Entrepreneurial experience [Novice (39%) = 1, Experienced = 0]	N/A	N/A	-0.162*	0.028	0.185*	-0.019	-0.012	-0.203*	**N/A**					
8. Number of employees	4.9868	8.01818	0.136	0.045	-0.232**	0.191*	-0.017	0.172*	-0.144	**N/A**				
9. Venture survival [Yes (91%) = 1, No = 0]	N/A	N/A	-0.030	-0.080	-0.093	-0.111	0.112	-0.079	-0.073	0.111	**N/A**			
10. Courage	59.934	0.042	0.034	-0.110	-0.104	0.133	0.104	0.128	-0.009	0.065	-0.106	**0.832**		
11. PsyCap	5.05	0.478	0.065	-0.098	-0.165*	0.145	0.242**	0.192*	-0.126	0.141	-0.035	0.552**	**0.812**	
12. Satisfaction	26.211	5.115	0.081	0.012	-0.016	0.027	0.099	-0.003	-0.040	-0.040	-0.045	0.176*	0.302**	**0.869**


*^∗^*p* < 0.05; ^∗∗^*p* < 0.01; *N* = 152. Cronbach’s Alphas are reported on the diagonal.*

**FIGURE 1:**
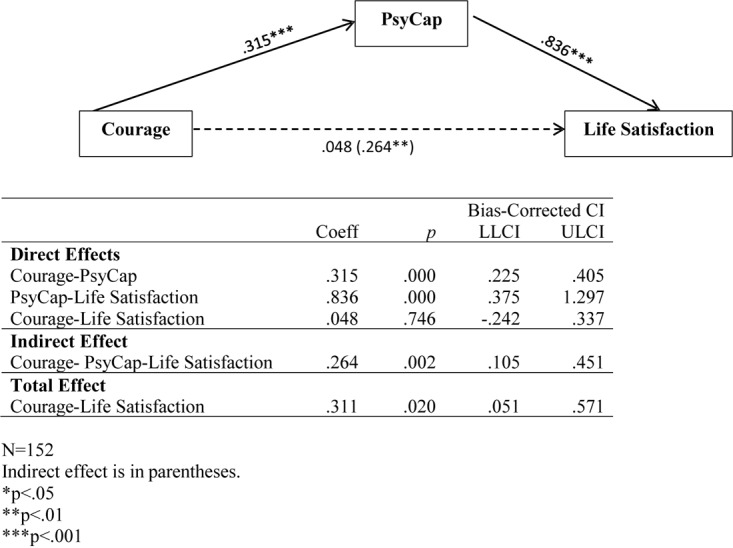
Mediation model results (5000 bootstrap samples).

We tested the hypothesized model using PROCESS v2.16.3 (Hayes, 2013). The nine control variables were included as covariates. As shown in Figure 1, courage has a significant direct effect on PsyCap, which in turn has a significant direct effect on life satisfaction. The direct effect of courage on life satisfaction is not significant. However, the indirect and total effects are significant. Thus, the effect of courage on life satisfaction was fully mediated by PsyCap (*Z* = 3.155, *p* < 0.01).

Common-method and single-source biases are common in one-time survey studies (Podsakoff et al., 2003). Thus, Harman’s single-factor test was conducted. Only 25.75% of the variance was explained by a single-factor, which is well below the 50% commonly used cutoff. Furthermore, we examined the item-level data using the common latent factor method in AMOS. Results showed poor model fit [Chi-square (405) = 1459.339, *p* = 0.000, CFI = 0.418, RMSEA = 0.131]. Common variance was 22.37%. Thus, these biases are unlikely.

Cross-sectional studies also run the risk of reverse causality. Thus, we ran five additional models (all the possible causal sequences among the three study variables) using both AMOS and PROCESS. With the exception of the hypothesized model and one alternative model (life satisfaction-PsyCap-courage), all of the other models showed poor fit. These two models also had the lowest AIC values (which can be used to compare non-nested models), representing the best fit (detailed results of these analyses are available from the authors).

## Discussion

This is the first study to explore courage in the context of entrepreneurship, and one of a few studies that examine entrepreneurs’ PsyCap. Entrepreneurs face extraordinary risks, challenges, and uncertainties. Positive psychological resources such as courage and PsyCap are necessary to start and sustain an entrepreneurial venture while maintaining life satisfaction and wellbeing.

A key finding is that PsyCap fully mediates the relationship between courage and life satisfaction in entrepreneurs. Thus, PsyCap may be an explanatory mechanism of this relationship. Contrary to anecdotes of fearless entrepreneurs who pursue entrepreneurship for the “thrill” or “rush” of taking extreme risks, courage seems to promote a more calculated approach to entrepreneurship. Specifically, Courage promotes hope, efficacy, resilience, and optimism, which share positive cognitive appraisals of success prospects based on motivation, effort, and perseverance (Luthans et al., 2007). Thus, courage does not promote reckless risk taking, or lead to unjustified satisfaction with an entrepreneurial lifestyle regardless of outcomes. Rather, courage promotes a positive and thoughtful approach to entrepreneurship that leads to life satisfaction based on intentional goal pursuit and achievement, effective deployment of effort and resources, and motivated persistence through obstacles and setbacks.

Limitations of this study include the small and homogenous sample, which limits external validity. Self-selection bias was also a concern, especially since most venture were still operating. However, venture survival was included as a control variable and was not significant. Common-method and single-source biases, as well as reverse-causality, are also potential validity threats as discussed earlier.

This study has several implications. Courage and PsyCap are malleable psychological resources that can be developed (Luthans et al., 2015; also see Youssef-Morgan and Petersen, 2019, for a comprehensive review of PsyCap development interventions). Furthermore, researchers, entrepreneurs and policymakers should adopt a more global perspective on entrepreneurial success. Financial measures are important, but they do not tell the full story. Many entrepreneurs are not “in it for the money,” but for a lifestyle of autonomy and intrinsic fulfillment. Societies that seek to promote entrepreneurship due to its economic benefits should not overlook its personal and subjective drivers and outcomes.

For future research, additional antecedents can be considered, including traits such as intelligence and personality, as well as more transient states such as moods and emotions. Contextual and environmental factors are also important and can represent boundary conditions in entrepreneurial contexts (Hmieleski and Baron, 2008, 2009; Hmieleski et al., 2013, 2015). Importantly, longitudinal and experimental research is necessary to further examine the causal relationships among the study variables. This study could also be replicated in other countries to examine cross-cultural consistencies and boundary conditions.

## Data Availability

The datasets for this study will not be made publicly available because this data set is the property of the authors.

## Ethics Statement

This study was carried out in accordance with the recommendations of Western IRB (WIRB), with written informed consent from all subjects. All subjects gave written informed consent in accordance with the Declaration of Helsinki. The protocol was approved by the Bellevue University internal IRB, as well as WIRB.

## Author Contributions

KB was responsible for data collection. Both authors contributed equally to formulating the conceptual framework, analyzing the data, and writing the manuscript.

## Conflict of Interest Statement

The authors declare that the research was conducted in the absence of any commercial or financial relationships that could be construed as a potential conflict of interest.
